# Genome-wide Identification and Characterization of FCS-Like Zinc Finger (FLZ) Family Genes in Maize (*Zea mays*) and Functional Analysis of *ZmFLZ25* in Plant Abscisic Acid Response

**DOI:** 10.3390/ijms22073529

**Published:** 2021-03-29

**Authors:** Shunquan Chen, Xibao Li, Chao Yang, Wei Yan, Chuanliang Liu, Xiaoyan Tang, Caiji Gao

**Affiliations:** 1 Guangdong Provincial Key Laboratory of Biotechnology for Plant Development, School of Life Sciences, South China Normal University, Guangzhou 510631, China; 2018010158@m.scnu.edu.cn (S.C.); 2018022421@m.scnu.edu.cn (X.L.); 20173029@m.scnu.edu.cn (C.Y.); 20185068@m.scnu.edu.cn (W.Y.); 2019010165@m.scnu.edu.cn (C.L.); 2 Shenzhen Institute of Molecular Crop Design, Shenzhen 518107, China

**Keywords:** ABA signaling, abiotic stresses, FLZ-domain protein, maize, *ZmFLZ*, *ZmKIN10*

## Abstract

FCS-like zinc finger family proteins (FLZs), a class of plant-specific scaffold of SnRK1 complex, are involved in the regulation of various aspects of plant growth and stress responses. Most information of *FLZ* family genes was obtained from the studies in *Arabidopsis thaliana*, whereas little is known about the potential functions of *FLZs* in crop plants. In this study, 37 maize *FLZ* (*ZmFLZ*) genes were identified to be asymmetrically distributed on 10 chromosomes and can be divided into three subfamilies. Protein interaction and subcellular localization assays demonstrated that eight typical ZmFLZs interacted and partially co-localized with ZmKIN10, the catalytic α-subunit of the SnRK1 complex in maize leaf mesophyll cells. Expression profile analysis revealed that several *ZmFLZs* were differentially expressed across various tissues and actively responded to diverse abiotic stresses. In addition, ectopic overexpression of *ZmFLZ25* in *Arabidopsis* conferred hypersensitivity to exogenous abscisic acid (ABA) and triggered higher expression of ABA-induced genes, pointing to the positive regulatory role of ZmFLZ25 in plant ABA signaling, a scenario further evidenced by the interactions between ZmFLZ25 and ABA receptors. In summary, these data provide the most comprehensive information on FLZ family genes in maize, and shed light on the biological function of *ZmFLZ25* in plant ABA signaling.

## 1. Introduction

The first FLZ-domain containing protein MEDIATOR OF ABA-REGULATED DORMANCY 1 (MARD1) was identified from senescence related enhancer-trapping and found to be implicated in abscisic acid (ABA)-mediated seed dormancy in *Arabidopsis* [1,2]. MARD1 possesses a novel zinc finger domain that shares high signature motif similarity with MYM-type zinc finger containing FCS sequence motif [2]. Subsequently, this domain was found to be exclusively existed in plants and designated as the FCS-Like Zinc finger (FLZ) domain or Domains of Unknown Function 581 (DUF581), which consists of around 70 amino acid residues harboring an identical CX_2_CX_17-19_FCSX_2_C motif [3,4]. Proteins containing this domain are named as FLZ family proteins, which are found in all taxa except algae [3,4], and are extensively involved in plant growth and stress responses [1,5,6,7,8].

The *Arabidopsis* genome encodes 18 FLZ proteins [3,9]. Gene expression analysis revealed diverse expression patterns of *AtFLZs*, and most of them were found to be highly expressed in reproductive organs, such as flower and silique, indicating potential specific functions of *AtFLZ* genes in regulating the development of these organs [4,9]. Interestingly, the highest expression of 17 *AtFLZs* occurs in 26 days old rosette, a prerequisite stage of flower bud appearance, indicating the potential important roles of *AtFLZ* genes in vegetative-to-reproductive phase transition [4]. In addition, *AtFLZ* genes were found to be highly responsive to plant hormone and nutritional cues, as well as various abiotic stresses, such as ABA, nitrogen starvation and high concentration of salt [4]. These studies highlight the potential roles of *FLZ* family genes during plant growth and stress response. Overexpression of *AtFLZ4/IRM1 (Increased Resistance to Myzus persicae 1)* increases the resistance to aphid attack, but leads to a significant reduction in plant growth in Arabidopsis [5]. Ectopic expression of a salt-induced wheat *FLZ* gene *TaSRHP* in *Arabidopsis* results in enhanced tolerance to salt and drought stresses [6]. In addition, *AtFLZ* genes were also found to be involved in the ABA-mediated seed dormancy and seedling growth [1,2,7]. Both *AtFLZ6* and *AtFLZ10* knock-down *Arabidopsis* mutants display higher sensitivity to ABA-repressed root elongation and lower tolerance to osmotic stress [7,8], albeit the underlying molecular mechanisms remain elusive.

Protein-protein interaction screening by yeast-two hybrid (Y2H) assay using AtFLZs as baits has successfully identified several FLZ-interacting proteins in *Arabidopsis* including the well-characterized energy-sensor SnRK1 kinase [4,9,10]. SnRK1 is a heterotrimeric kinase complex consisting of α catalytic and βγ regulatory subunits [11,12,13]. Further studies revealed that the FLZ domain enables the interactions between FLZ proteins and the catalytic subunit SnRK1α, whereas the intrinsically disordered region (IDR) in the N terminus facilitates their interactions with the β and γ subunits of SnRK1, suggesting a function of FLZ protein as the scaffold of SnRK1 complex [9,14]. However, the molecular mechanisms of how FLZs modulate the activity of SnRK1 complex remain elusive. SnRK1 was found to repress the transcription of sugar-inducible FLZ genes (such as *FLZ2*, *FLZ3*, and *FLZ8*) but induce the expression of sugar-inducible *FLZ9* in *Arabidopsis* [4], suggesting the complicated connection of *FLZs* with the SnRK1 signaling cascade at the transcriptional level. More interestingly, one recent report illustrated that two carbon starvation-induced *FLZ* genes, *FLZ6* and *FLZ10*, work as repressors of SnRK1 signaling in Arabidopsis [7]. Knock-down of these two genes resulted in an increase in the protein level of SnRK1α1 and a concomitant enhancement of the SnRK1 activity. In line with these findings, the *flz6* and *flz10 Arabidopsis* mutants were found to display similar phenotype to SnRK1α1 overexpression plants with compromised growth under normal growth condition [7].

Maize is an important food crop and a source of industrial materials worldwide [15]. Although the genome sequencing of maize has been obtained [16,17,18], there is still a lack of relevant research on the *FLZ* family genes in maize. In this work, we clarified the bioinformatics characteristics of *ZmFLZs* genes as well as their tissue-specific and stress-responsive expression patterns and protein subcellular localizations in maize. Furthermore, the potential protein-protein interactions between ZmFLZs and ZmSnRK1α were also investigated. Finally, the biological role of *ZmFLZ25* in ABA signaling was analyzed in the ectopic expressed *Arabidopsis*. These studies provide a useful reference for the further functional analysis of the FLZ family genes in maize.

## 2. Results

### 2.1. Identification of FLZ Family Genes in Maize Genome

**Figure 1:**
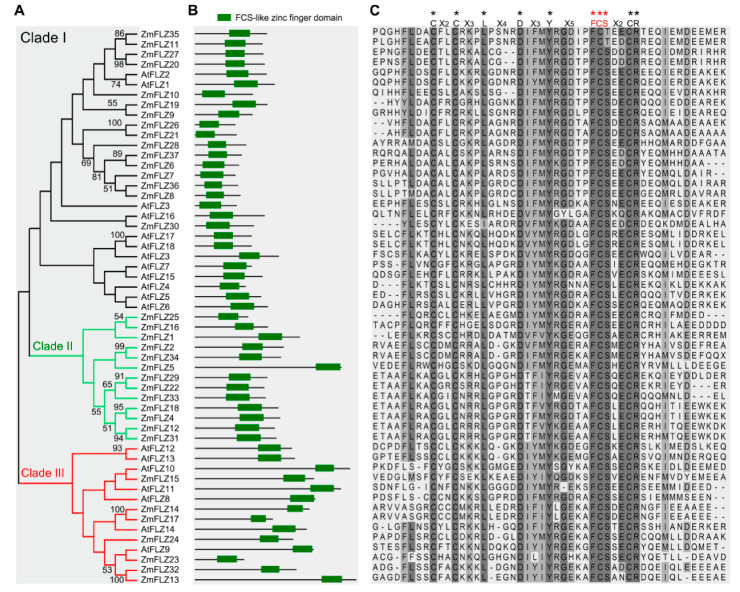
Phylogenetic tree and domain feature of FLZs in maize and *Arabidopsis*. (**A**) Phylogenetic tree of FLZ proteins in maize and *Arabidopsis*. The phylogenetic tree was generated using the Neighbor joining method in the MEGA-X software with 1000 replicates bootstrap analysis and complete deletion. The numbers at the nodes indicate the bootstrap values, with cutoff no less than 50. (**B**) Diagrams of the FLZ domain in the FLZ proteins. The conserved FLZ domains were predicted with NCBI Batch-CD and marked with green boxes. (**C**) Protein sequences alignment of the FLZ domain. ClustalW was used for protein alignment. The conserved amino acids in FLZ domains were highlighted and marked with asterisks.

The amino acid sequences of FLZ domain from AtFLZ proteins were used as queries to search the homologous proteins in maize genome against the B73 genome in MaizeGDB (https://www.maizegdb.org/; accessed on 1 September 2019). In total, 37 independent FLZ proteins (ZmFLZs) were identified in maize genome (B73) (Figure 1A and Table S1). These 37 putative ZmFLZs were then submitted to NCBI Batch-CD and Pfam to confirm the existence of FLZ domain (Figure 1B), and their corresponding coding genes were orderly named *ZmFLZ1* to *ZmFLZ37* in accordance with their locations on the chromosomes of maize (Table S1). Detail information regarding the 37 *ZmFLZ* genes (gene ID and DNA attributes, such as gene locus, open reading frame, and number of exons) as well as their encoded proteins (protein molecular weight, isoelectric point, and predicted subcellular localization) are listed in Table S1. These 37 *ZmFLZ* genes are unevenly distributed on the 10 chromosomes, and the highest numbers of them are found on chromosomes 2, 4 and 5 (7 each), while chromosomes 6, 7 and 8 only contain a single *ZmFLZ* gene (Table S1 and Figure S1). Subsequent sequence analysis showed diverse features of the 37 *ZmFLZ* genes in open reading frames, ranging from 273 (*ZmFLZ7*) to 1077 (*ZmFLZ13*) base pair, and most of them (29 out of 37) contain two exons (Table S1). In addition, the lengths of ZmFLZ proteins range from 90 to 358 amino acids with predicted molecular weights range from 9.6 to 36.45 kDa and isoelectric points from 4.38 to 11.33 (Table S1). All ZmFLZs are predicated to be soluble proteins without transmembrane domains and N-terminal signal peptide (Table S1).

### 2.2. Phylogenetic and Structural Analysis of FLZ Proteins

To investigate the phylogenetic relationship between the *FLZ* genes in maize and *Arabidopsis*, a phylogenetic tree was constructed based on their full-length protein sequences using MEGA-X software according to Neighbor joining method (Figure 1A). As shown in Figure 1A, 55 FLZ proteins (37 ZmFLZs and 18 AtFLZs) are naturally divided into 3 major clades, designated as I, II and III, consisting of 28, 13 and 14 members, respectively. Except the smallest subfamily II that exclusively contains maize FLZs, the other two clades contain FLZs from both maize and *Arabidopsis* (Figure 1A). However, most of the FLZ members are tended to be clustered in species-specific clades (Figure 1A). The FLZ domain is predicted to be the only conserved functional domain in maize and *Arabidopsis* FLZ proteins (Figure 1B). A multiple sequence alignment of the FLZ domains revealed that CX_2_CX_3_LX_4_DX_3_YX_5_FCSX_2_CR is a highly conserved motif in the FLZ domain region among those FLZ proteins in maize and *Arabidopsis* (Figure 1C). Collectively, the above information suggests that the main characteristics of FLZ proteins in maize and *Arabidopsis* were formed before the divergence of monocots and dicots, and then evolved separately in a species-specific manner.

### 2.3. Tissue Specific Expression Pattern of ZmFLZ Genes

**Figure 2:**
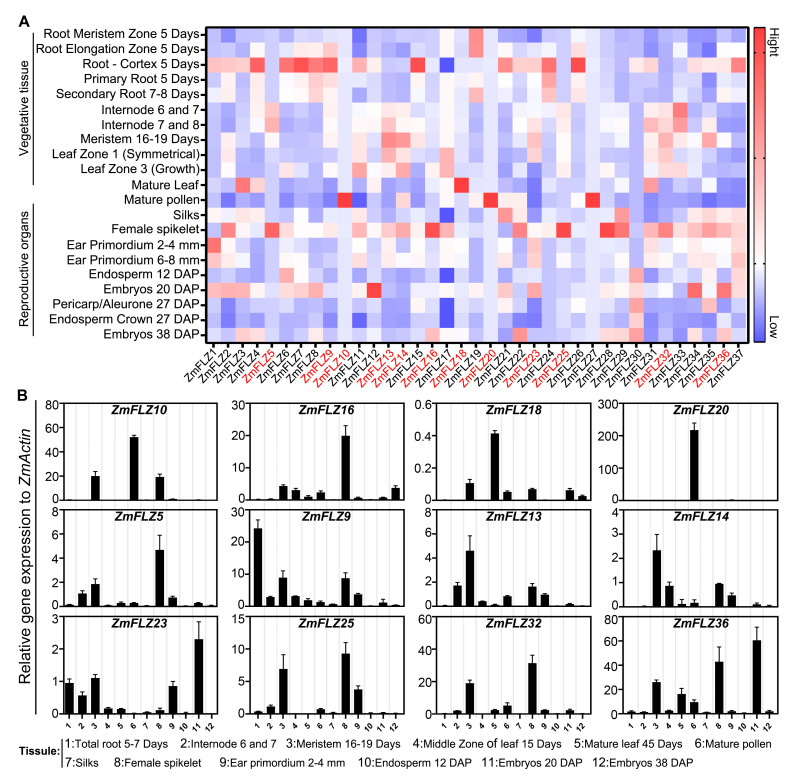
Spatial expression patterns of *ZmFLZs*. (**A**) Heat map of the gene expression of *ZmFLZs* in various tissues or developmental stages. The data were downloaded from the qTeller of MaizeGDB (https://qteller.maizegdb.org/; accessed on 1 September 2019). The expression levels as indicated by Fragments Per Kilobase of exon model per Million mapped fragments (FPKM) were shown from low (blue) to high (red) for each gene. (**B**) Tissue-specific expression patterns of *ZmFLZs* by qRT-PCR assay. The *x*-axis represents different tissues or organs. The *y*-axis shows the relative gene expression levels normalized to *ZmActin*. Data represent the mean ± SEM from three independent experiments. DAP, day after pollination.

Gene expression pattern provides important clues for understanding potential gene function. To clarify the spatial expression profiles of *ZmFLZ* genes, we first retrieved the publicly available RNA-seq data from maize the MaizeGDB database (https://www.maizegdb.org/; accessed on 1 September 2019), and constructed an expression map of all the 37 *ZmFLZ* genes in 21 different tissues, including 11 vegetative organs (like root, internode, and leaf) and 10 reproductive organs (like ear primordium, spikelet, silk, mature pollen, pericarp, endosperm and embryo). As shown in Figure 2A, most of the *ZmFLZ* genes were ubiquitously expressed in various tissues, but the expression levels in different tissues varied largely. Some *ZmFLZ* genes showed highly tissue-specific expression patterns, for instance, *ZmFLZ12* is highly expressed in embryos 20 days after pollination (DAP), whereas four *ZmFLZ* genes including *ZmFLZ5, ZmFLZ16, ZmFLZ25* and *ZmFLZ28* are preferentially expressed in female spikelet. In addition, *ZmFLZ10*, *ZmFLZ20,* and *ZmFLZ27* are preferentially expressed in mature pollen, while *ZmFLZ18* is highly expressed in mature leaf (Figure 2A). To experimentally verify the expression profiles of selected *ZmFLZs*, we firstly tested the expression data of four *ZmFLZs* (*ZmFLZ10*, *ZmFLZ16*, *ZmFLZ18* and *ZmFLZ20*), all of which have highly specific expression pattern. The results of quantitative real-time PCR (qRT-PCR) showed that these four selected *ZmFLZ* genes displayed peak expression in specific tissues like *ZmFLZ10* and *ZmFLZ20* in mature pollen, *ZmFLZ16* in female spikelet as well as *ZmFLZ18* in mature leaf (Figure 2B), which were largely consistent with results obtained from the publicly available RNA-seq data (Figure 2A). Secondly, according to the phylogenetic analysis, we selected and checked the transcript abundance of eight representative *ZmFLZ* genes (*ZmFLZ9* and *ZmFLZ36* from clade I, *ZmFLZ5* and *ZmFLZ25* from clade II, *ZmFLZ13*, *ZmFLZ14*, *ZmFLZ23*, and *ZmFLZ32* from clade III) in 12 different tissues or developmental stages. The results of qRT-PCR showed that the expression patterns of these eight selected *ZmFLZ* genes were generally in agreement with the data of RNA-seq analysis. For example *ZmFLZ36* exhibited the highest expression in embryo 20 DAP (Figure 2B). Relative high expression level of *ZmFLZ13* and *ZmFLZ14* were observed in vegetative meristem of 16–19 days old maize seedling.

### 2.4. Subcellular Localizations of Eight Typical ZmFLZ Proteins and Their Interactions with ZmKIN10

**Figure 3:**
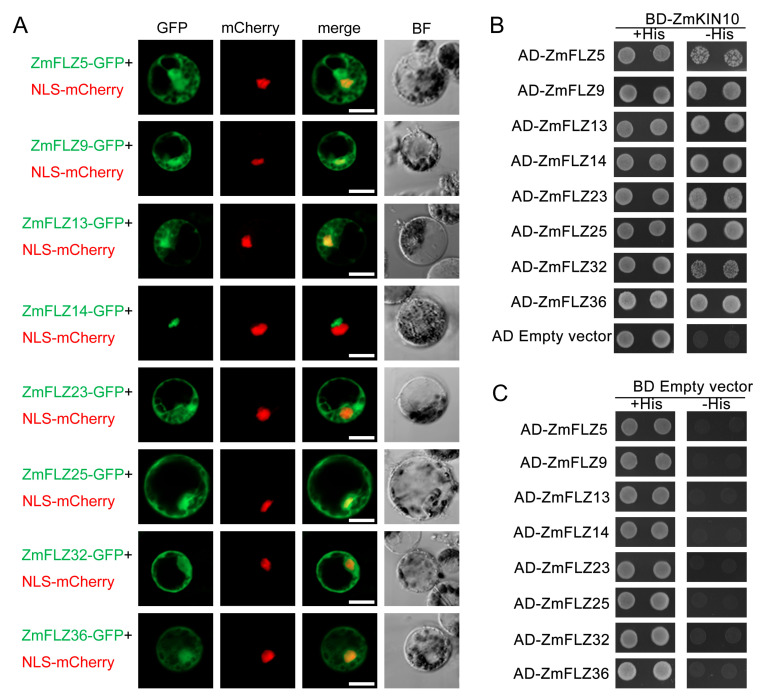
Subcellular localization of the eight selected ZmFLZs and ZmFLZs interaction with ZmKIN10 by Y2H assay. (**A**) ZmFLZs-GFP fusion constructs were used to determine the subcellular localization of ZmFLZs in the protoplast cells isolated from maize leaves. NLS-mCherry was used as a nuclear marker. Fluorescent images of GFP and mCherry were captured with a confocal laser scanning microscopy and shown in green and red, respectively. Scale bars = 25 μm. (**B**,**C**) Yeast two hybrid analysis of the binary interactions between the eight selected typical ZmFLZs and ZmKIN10.

Bioinformatics analyses indicate that ZmFLZs are potentially targeted to several subcellular compartments (Table S1). To experimentally determine the subcellular localization of ZmFLZs from different subfamilies, eight typical ZmFLZs were randomly selected from three different clades according to the above phylogenetic analysis. These eight selected members include ZmFLZ9 and ZmFLZ36 from clade I, ZmFLZ5 and ZmFLZ25 from clade II, ZmFLZ13, ZmFLZ14, ZmFLZ23, and ZmFLZ32 from clade III. To this end, the full-length coding sequences of the eight selected typical *ZmFLZs* were fused in-frame to the N-terminus of *Green Fluorescent Protein* (*GFP*) driven by the *UBQ10* promoter. Then the resulting fusions were co-expressed with the nuclear marker NLS-mCherry in the maize mesophyll protoplasts. Confocal microscopic analysis showed that GFP-fused ZmFLZ5, 9, 13, 23, 25, 32, and 36 localized in both cytoplasm and nucleus; while the fluorescent signal of ZmFLZ14-GFP was near to but not co-localized with NLS-mCherry (Figure 3A). The results were, in general, consistent with the bioinformatics prediction of the subcellular localization of ZmFLZs (Table S1).

**Figure 4:**
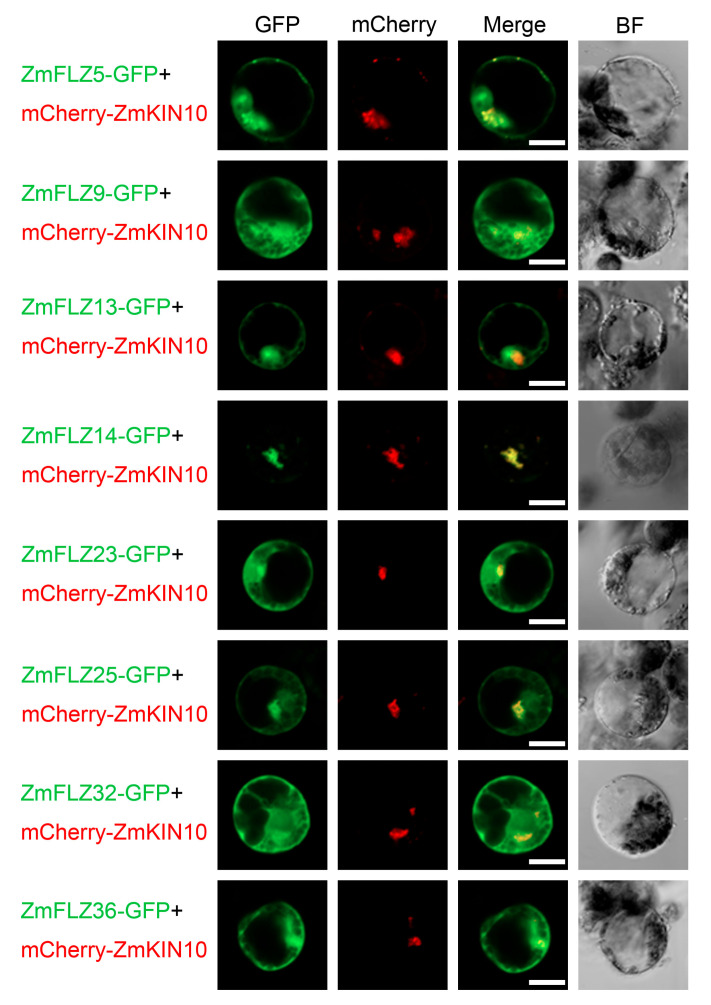
Co-localization analysis of ZmFLZs and ZmKIN10 in maize leaf protoplast cells. Co-expression of mCherry-ZmKIN10 and the GFP fused ZmFLZs in protoplasts followed by confocal imaging. Fluorescent images of GFP and mCherry were captured with a confocal laser scanning microscopy and shown in green and red, respectively. Scale bars = 25 μm.

Previous reports demonstrated that FLZ proteins could interact with the α-subunit of SnRK1 kinase complex and function as the scaffolding proteins of SnRK1 complex in *Arabidopsis* [7,8]. To verify whether ZmFLZs also interact with ZmSnRK1 complex in maize, we used yeast two-hybrid (Y2H) assay to detect the interactions between these eight selected ZmFLZs and the maize SnRK1 subunit ZmKIN10. To this end, the full-length ZmFLZs were fused to GAL4 activation domain (AD-ZmFLZ) and ZmKIN10 was fused to DNA binding domain (BD-ZmKIN10) vectors, respectively. The AD-ZmFLZs were separately co-transformed with the BD-ZmKIN10 into yeast cells, and the interaction of them was evaluated based on the growth of yeast cells on SD/-Trp/-Leu/-His medium plates. As shown in Figure 3B, all the yeast cells harboring BD-ZmKIN10 and AD-ZmFLZ pairs could well grow on the SD/-Trp/-Leu/-His selective medium, suggesting that these ZmFLZs proteins could directly interact with ZmKIN10 in yeast. In order to further explore the functional connection of ZmFLZs and ZmKIN10, ZmFLZs-GFP and ZmKIN10-mCherry were co-transformed into maize leaf protoplasts to observe their subcellular localizations (Figure 4). The obtained results showed that the fluorescent signal of mCherry-ZmKIN10 partially overlapped with the GFP tagged ZmFLZs on the cytoplasmic aggregates (Figure 4), whose shape is obviously different from the smooth and round shape of the nucleus as labeled by the nuclear marker NLS-mCherry (Figure 3). Interestingly, the non-nuclear localized ZmFLZ14-GFP showed a complete colocalization with mCherry-ZmKIN10 when co-expressed in the same cell. These results indicate the close association of these FLZ proteins with ZmKIN10 inside maize leaf mesophyll cells.

### 2.5. Stress-Responsive Expression Profiles of ZmFLZ Genes

**Figure 5:**
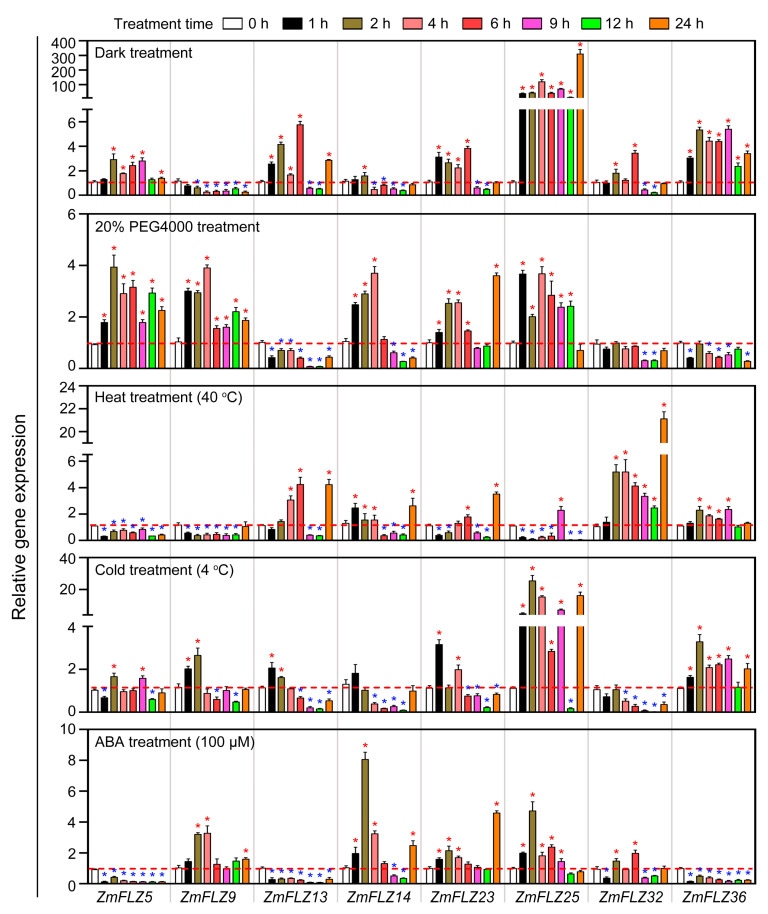
Expression pattern of *ZmFLZs* in response to stress treatments. Seedlings of B73 at V3 stage (three-leaf-stage, around 15 days after germination) were separately subjected to darkness, drought (20% PEG4000), heat (40 °C), cold (4 °C), and 100 μM ABA treatments for 24 h. The leaves were harvested for gene expression analysis by qRT-PCR at 0 h, 1 h, 2 h, 4 h, 6 h, 9 h, 12 h, and 24 h, respectively. Seedlings grown under the normal conditions were harvested at the same time points and used as the controls. Relative gene expression levels of *ZmFLZs* were calculated as the ratio of “treatment” to “control” after being normalized against *ZmActin*. The mean ± SEM from three independent experiments were shown for each treatment. Multiple *t*-test was included to estimate the differences between the expression levels under stresses and untreated (0 h) conditions for each *ZmFLZ* gene. The red and blue asterisks indicated significantly enhanced and repressed expression under stresses, respectively (*p*-value < 0.05).

Maize is frequently challenged by abiotic stresses such as shade, drought, and high or low temperature in the field. Recent studies have suggested that *AtFLZ* genes are widely involved in signaling and response to abiotic stimuli [4]. To study the potential roles of *ZmFLZ* genes in response to abiotic stresses, we next performed qRT-PCR analysis for the aforementioned eight selected *ZmFLZ* genes in maize seedlings under multiple abiotic stress conditions, including dark, drought (20% PEG4000), heat (40 °C), cold (4 °C) and ABA (100 μM) (Figure 5). All of these stresses seriously impaired the normal growth of maize seedling and induced the expression of stress-responsive genes (Figure S2). As shown in Figure 5, the transcription of the eight *ZmFLZ* genes illustrated dynamic and active responses to different abiotic stresses. In the dark treatment, the expressions of *ZmFLZ25* and *ZmFLZ36* were induced 3~100-fold, while the expression level of *ZmFLZ9* was significantly repressed throughout the time course. The expression of *ZmFLZ5*, *ZmFLZ13*, *ZmFLZ23* and *ZmFLZ32* were unregulated during the early time points, and then down-regulated at the later time points, while *ZmFLZ14* was down-regulated from 4 h to 12 h after dark exposure. PEG-mimicked drought stress also had strong effect on the expression of these eight examined *ZmFLZ* genes, in which *ZmFLZ13, ZmFLZ32* and *ZmFLZ36* were repressed but the expression levels of *ZmFLZ5*, *ZmFLZ9*, *ZmFLZ23* and *ZmFLZ25* were elevated at most of the checked time points. Interestingly, the expression of *ZmFLZ14* was increased after PEG treatment for 1 h to 4 h, and then decreased subsequently.

For the heat treatment, *ZmFLZ5*, *ZmFLZ9* and *ZmFLZ25* were markedly downregulated at most of the checked time points. In contrast, the expression of *ZmFLZ32* was significantly increased, with >20-fold increase after heat treatment for 24 h. Other four *ZmFLZ* genes (*ZmFLZ13,* *14*, *23* and *36*) were differentially expressed at specific time points during heat treatment (Figure 5). Upon the cold treatment, *ZmFLZ25* and *ZmFLZ36* showed increased expression patterns at almost all checked time points except for the stage of treatment for 12 h. The expression levels of *ZmFLZ13*, *ZmFLZ14* and *ZmFLZ32* were slightly increased and then significantly decreased from 6 h after cold treatment. *ZmFLZ23* was obviously upregulated at 1 h and 4 h points and then downregulated afterwards (Figure 5). Finally, we evaluated the responses of these eight *ZmFLZ* genes to ABA, the most important stress-protective phytohormone in plants [19]. *ZmFLZ5*, *ZmFLZ13* and *ZmFLZ36* were dramatically suppressed by ABA treatment, while the expressions of other five *ZmFLZs* were mostly upregulated at the early stages after ABA treatment (Figure 5). These results indicate that different *ZmFLZs* might have diverse roles in stress response to various stresses.

### 2.6. Ectopic Expression of ZmFLZ25 in Arabidopsis Confers Plant the Sensitivity to ABA Treatment

**Figure 6:**
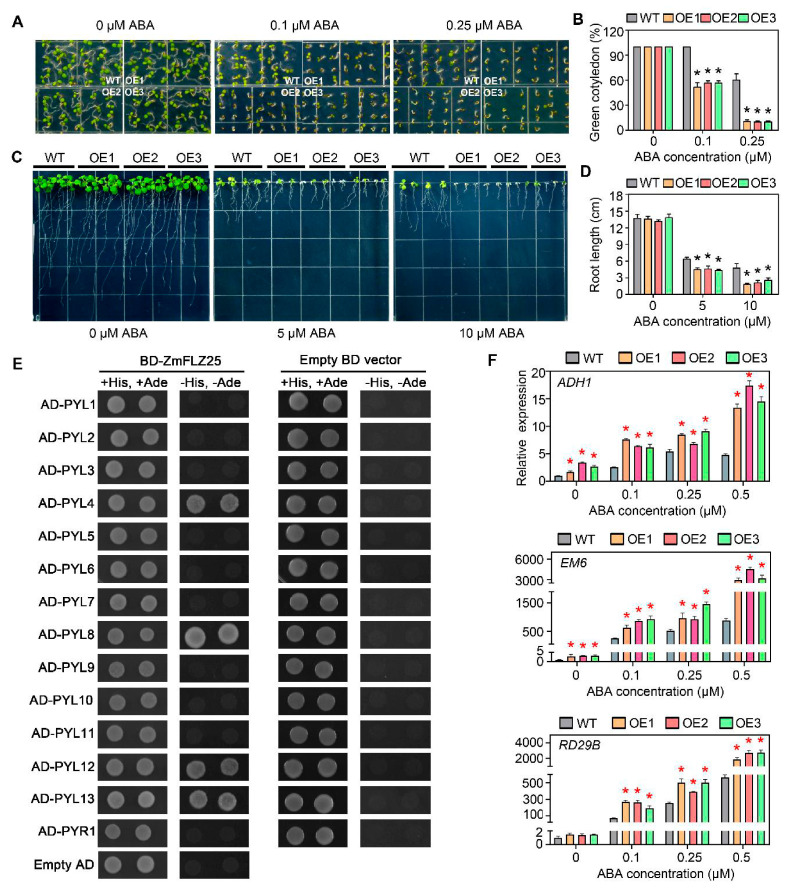
Overexpression of *ZmFLZ25* in *Arabidopsis* resulted in the hypersensitivity of plant to ABA. (**A**) Overexpression expression of *ZmFLZ25* in *Arabidopsis* confers plant sensitivity to ABA 5. days after imbibition. The OE1, OE2 and OE3 represent three different *ZmFLZ25*-overexpression lines. (**B**) The cotyledon greening rate of WT and OE lines were counted after ABA treatments. Approximately approximately100 seeds were counted for each genotype at 5 days of growth on 1/2 Murashige & Skoog (MS) medium plate, data represents three biologically replicates. (**C**) Ectopic expression of *ZmFLZ25* in *Arabidopsis* resulted in enhanced root growth inhibition in response to ABA treatment. WT and overexpression seedlings were grown on 1/2 MS plate for 2 days and subsequently transferred to plates containing 5 μM and 10 μM ABA for 7 days. (**D**) Statistics of the root length of WT and OE lines after ABA treatments corresponding to (**C**). (**E**) Protein interactions between ZmFLZ25 and *Arabidopsis* ABA receptors by yeast two hybrid assay. (**F**) The expression of ABA-responsive genes (*ADH1*, *EM6*, *RD29B*) (*y*-axis) in *Arabidopsis* under treatments with different concentrations of ABA (*x*-axis) in WT and OE lines. The relative gene expression levels were normalized to the internal gene *AtUBQ*. All the data are the mean ± SEM from three independent experiments. The asterisks indicated significantly differences between WT and OE lines as determined by *t*-test (*p*-value < 0.05).

*ZmFLZ25* belongs to the maize-specific subfamily II (Figure 1A) and is highly responsive to various stress treatments (Figure 5). Therefore, we tried to study the functional role of this gene in plant stress response. To this end, *ZmFLZ25-GFP* construct driven by the UBQ10 promoter was introduced into *Arabidopsis* (Columbia-0). We totally obtained 12 individual T_3_ homozygous transgenic lines.Three independent overexpression lines (OE1, OE2 and OE3) with higher expression level of *ZmFLZ25* as confirmed by qRT-PCR assay (Figure S3) were chosen for further study. Compared with the wild-type (WT) plant, these OE lines showed no obvious phenotype under regular growth condition. However, when directly sowed on the 1/2 MS plates containing different concentrations of ABA, the OE lines displayed enhanced sensitivity to ABA-mediated inhibition of seedling establishment when compared to the WT (Figure 6A,B). To further compare the sensitivity of these plants to ABA, we transferred the 2-d-old plants of WT and OE lines to the fresh 1/2 MS plates supplemented with 0, 5 and 10 μM ABA for additional 7 days growth. As shown in Figure 6C,D, ABA treatment seriously inhibited the primary root growth of *Arabidopsis* seedlings, and such an effect was more obvious seen in the roots of *ZmFLZ25-OE* seedlings that were significantly shorter than wild-type seedlings (*p*-value ≤ 0.05). These data demonstrated that the *ZmFLZ25-OE* lines are hypersensitive to ABA treatment. To explore the possible molecular mechanisms underlying this ABA hypersensitive phenotype, we examined the possible protein interactions between ZmFLZ25 and *Arabidopsis* ABA-responsive signaling components. To this end, we checked the protein interactions between ZmFLZ25 and the 14 ABA receptors [19,20,21]. Interestingly, we found that the ZmFLZ25 could interact with several ABA receptors including PYL4, PYL8, PYL12 and PYL13 (Figure 6E), indicating a direct connection between ZmFLZ25 and ABA signaling. We also analyzed the expression of ABA-responsive genes *ADH1*, *EM6* and *RD29B* in *ZmFLZ25-OE* plants. The obtained results showed that gene expression level of these ABA-responsive marker genes was more obviously enhanced in *ZmFLZ25-OE* plants than that in the wild-type seedlings in response to ABA treatment (Figure 6F). Taken together, these results suggest that ZmFLZ25 interacts with the ABA receptors and might function as a positive regulator in ABA signaling during seedling establishment and growth.

## 3. Discussion

In this study, 37 *ZmFLZ* genes were identified in the maize genome via a homology-search method. These 37 ZmFLZ proteins can be divided into three subfamilies with highly conserved FLZ domain but significantly different protein length. Moreover, we found that *ZmFLZ* genes have a varied expression patterns with some organ-specific induction trend, while the relative higher expression of most *ZmFLZ* genes were observed in 5-day-old root cortex and female spikelets (Figure 2A). The qRT-PCR analysis of twelve typical *ZmFLZ* genes showed that three *ZmFLZs*, including *ZmFLZ10*, *ZmFLZ16* and *ZmFLZ20*, display a specifically high expression in reproductive organs, while the remaining nine selected *ZmFLZs* show high expression levels in vegetative meristem and internodes as well as endosperm (20 DAP) (Figure 2B). These results suggested the differential and specific roles of *ZmFLZ* family members during maize growth and organ or tissue development. In supporting this noting, one root expressed *FLZ* gene *ZmFLZ14* was recently found to be well associated with maize lateral root branching frequency under well-water conditions [22], highlighting its potential roles in the control of root architecture in maize. In *Arabidopsis*, it has been shown that several *AtFLZs* exhibited higher expression levels in the reproductive organs, such as, flower and/or silique [4], and overexpression of the *FLZ* gene *AtFLZ4/IRM1* led to delayed bolting time and smaller size of siliques and less production of seeds in *Arabidopsis*, indicating an important role of FLZ family genes in plant reproduction [5]. In future, it will be interesting to dissect the roles of those *ZmFLZs* specifically expressed in reproductive organs in corn development and production.

Previous studies have well established that expression of *FLZ* genes is differentially regulated by hormones and environmental cues, as well as cellular energy level, suggesting their implication in plant stress signaling or response [4,6,9]. For example, expression of the wheat *FLZ* gene *TaSRHP* is salt-induced, and ectopic overexpression of this gene in *Arabidopsis* conferred enhanced tolerance to salt and drought stresses [6]. Furthermore, one recent report showed that knock-down of two ABA-induced *FLZ* genes, *FLZ6* and *FLZ10* resulted in the hypersensitive of seedlings to ABA and osmotic stresses [7,8]. In this study, we found that eight *ZmFLZ* genes are highly responsive to abiotic stresses, including dark, drought, heat, cold and ABA (Figure 5). For instance, the expression level of *ZmFLZ25* dramatically increased in maize seedlings under dark, cold or ABA treatments. These results indicated that some *ZmFLZ* genes might be involved in stress response to adverse environmental conditions. But up to now, how *FLZs* are involved in plant stress signaling and response remains largely unknown. For instance, *ZmFLZ25* showed the most notable response to all of the five stresses, among them, heat treatment significantly repressed its expression, whereas dark, drought, cold and ABA treatments up-regulated its expression (Figure 5). To clarify the potential roles of *ZmFLZ25* in plant stress response, we generated *ZmFLZ25-OE* transgenic *Arabidopsis* plants and found that *ZmFLZ25-OE* plants displayed higher sensitivity to ABA treatment (Figure 6), pointing to the positively regulatory role of *ZmFLZ25* in plant ABA signaling. Consistent with the roles of FLZs in the positive regulation of plant ABA signaling, knock-down of the FLZ family gene *MARD1* in *Arabidopsis* conferred resistance to ABA-regulated seed dormancy, and the mutant seeds germinated faster comparing with the WT seeds in the medium supplemented with exogenous ABA [2]. However, the direct link between FLZs and ABA signaling remain totally unclear. Here, we showed that the maize FLZ member ZmFLZ25 could directly interact with several ABA receptors including PYL4, PYL8, PYL12 and PYL13. Considering the positive regulatory role of ZmFLZ25 in *Arabidopsis* ABA response, it is possible that binding of ZmFLZ25 might result in protein stabilization and less degradation of ABA receptors, or might mimic the FLZ function as a scaffold of SnRK1 kinase complex to enable the enhanced interactions between PYLs and PP2Cs, thereby activating ABA signaling pathway. Future work is needed to dissect the exact roles of FLZ family in plant ABA signaling.

Protein-protein interaction network analysis showed that FLZ proteins might interact with several functional proteins that are involved in well-characterized stress signaling pathways such as SnRK1, TOR and MPK signaling [4,9,10]. In this work, we demonstrated that ZmFLZs interacted with ZmKIN10 in the yeast-two-hybrid assay (Figure 3B,C) and found that several ZmFLZs partially co-localized with ZmKIN10 in maize cells (Figure 4). Although, these results are largely consistent with the results of FLZ-SnRK1 interactions in *Arabidopsis* [7,8,9], it is worth noting that the subcellular compartment where ZmSnRK1 and ZmFLZs co-localized with each other in maize cells seems to be the cytoplasmic aggregates rather than the nucleus (Figure 4). Interestingly, the *Arabidopsis* KIN10 has been reported to target to diverse cellular compartments, such as the cytoplasm and nucleus [23,24], some unknown puncta [25,26], the Golgi [27], chloroplasts [28], and the endoplasmic reticulum (ER) [14,29]. Moreover, a recent report has demonstrated that AtKIN10 interacted with AtFLZs in onion epidermal cells, and the fluorescent signals of their interaction site were observed in cytoplasmic foci, which were further confirmed as the ER associated with the nucleus [14]. Based on the evolutionary conservation of FLZ-SnRK1 interaction in different plant species and the localization pattern we observed in this study, we suspected that the cytoplasmic aggregates where ZmFLZs and ZmKIN10 co-localized might be a portion of ER near the nucleus in maize cells. In future, more accurate experiments are needed to further clarify the subcellular distributions of ZmFLZ and ZmSnRK1 in maize.

It is clear that SnRK1 kinase complex is the cellular energy sensor, which can be activated by very diverse abiotic and biotic stress conditions that directly or indirectly cause an energy deficit [11,30,31]. Although FLZs function as the scaffold of SnRK1 complex, how FLZs affect SnRK1 signaling remains elusive. One recent study has revealed that FLZ6 and FLZ10 act as suppressors of SnRK1 to regulate *Arabidpsis* growth and the responses to ABA and sugar by modulating the protein abundance of SnRK1 [7], but the underling molecular mechanism remains elusive. So in the future, more genetic, molecular and physiological studies are needed to decipher how FLZs affect SnRK1 signaling to modulate plant stress response. As an important food, feeds, and industrial raw material, maize is often exposed to various abiotic stresses such as drought, extreme temperature, and shade, which might cause reduced or even no yield [32,33,34,35]. Further studies of the molecular functional roles of SnRK1-FLZ module in maize will be helpful for future applications of this conserved regulatory module in maize breeding to enhance stress-resistance.

## 4. Materials and Methods

### 4.1. Data Search and Analyses

Search of FLZ proteins in maize was done as described previously [36]. Briefly, the protein sequences of FLZ domain from AtFLZs were used as queries to run the BLAST program against the B73 reference genome (RefGen_v4) (https://www.maizegdb.org/; accessed on 1 September 2019) [37]. A self-BLAST of these sequences was also performed to remove redundant sequences, and then the remaining sequences were submitted to the Pfam search (https://pfam.xfam.org/; accessed on 15 October 2019) [38] and BLAST-based NCBI conserved domain search (https://www.ncbi.nlm.nih.gov/Structure/cdd/wrpsb.cgi; accessed on 26 November 2019) to further confirm the presence of the conserved FLZ domain, and the protein structures of them were visualized with the TBtools software [39]. Information about gene locus, chromosome location, nucleotide length, protein length, protein molecular weight, protein isoelectric point was also obtained for each *ZmFLZ* gene from maize database (https://www.maizegdb.org/; accessed on 1 September 2019). The distribution pattern of *ZmFLZ* genes on the chromosomes were visualized with a RCircos package in R. All the FLZ protein sequences from maize and *Arabidopsis* were aligned using ClustalW, and then the alignment result was imported in MEGAX to generate a phylogenetic tree using the Neighbor joining method with partial deletion and 1000 bootstraps.

### 4.2. Maize Growth Conditions and Treatments

Seeds of maize B73 were obtained from Institute of Germplasm Resources and Biotechnology, Jiangsu Academy of Agricultural Sciences, China. Seedling cultivation was performed according to the methods described previously [40]. Briefly, the surface-sterilized plump seeds were cultured in water in a growth chamber at 28 °C (light) /25 °C (dark) with a 14 h (light)/10 h (dark) photoperiod. After five days, the uniformed seedlings were selected and further cultured with 1/2 Hoagland (Phygene, Shenzhen, China) nutrient solution to the V3 stage. Then the seedlings were planted in the field and the different tissues at the indicated developmental stages were harvested to analyze the gene expression of *ZmFLZs*.

The stress treatments of maize seedlings were performed as previously described [41]. For each treatment, three uniformed maize plants of B73 at the V3 stage were used, and the untreated maize seedlings at the same time points were used as the control. For dark treatment, seedlings were kept in the full darkness. For drought stress, seedlings were root-cultured in nutrient solution containing 20% PEG4000 (Sangon Biotech, Shanghai, China). Seedlings were transferred to a 40 °C or 4 °C incubator respectively for heat and cold treatments. For ABA treatment, maize plants were root-soaked in the nutrient solution containing 100 μM ABA (Sangon Biotech, Shanghai, China). After 0 h, 1 h, 2 h, 4 h, 6 h, 12 h and 24 h treatments, leaf samples of these treated or control seedlings were harvested and frozen in liquid nitrogen for further RNA isolation.

### 4.3. Generation of ZmFLZ25 Overexpressed Arabidopsis and ABA Sensitivity Test

The BLAST search against the *Arabidopsis* database TAIR website (https://www.arabidopsis.org/; accessed on 8 February 2021) showed that ZmFLZ25 shares the highest similarity (59%) to AtFLZ4 (AT5G65040.1). To overexpress *ZmFLZ25*, the coding sequence (CDS) of *ZmFLZ25* was PCR-amplified and inserted into the binary vector pCAMBIA1300 to in-frame fuse with a *GFP* gene. The resulting *pCAMBIA1300-UBQ10-ZmFLZ25-GFP* construct was introduced into wild-type *Arabidopsis* (Col-0) plants by Agrobacteria-mediated floral dipping. The positive transgenic plants were selected on standard 1/2 MS (Murashige and Skoog) plates containing 50 μg/L Hygromycin B (Sangon Biotech, Shanghai, China) and the T_3_ homozygous transgenic lines were used for further analysis [42].

For ABA sensitivity test during seedling establishment, seeds were surface sterilized and stratified at 4 °C for 48 h in darkness before sowing on standard 1/2 MS plates supplemented with 0, 0.1 and 0.25 μM ABA. Then the plates were placed in a growth chamber at 22 °C under the 16 h (light)/8 h (dark) conditions for five days before photographing and recording the seedling growth rate following the procedure as described previously [43]. For ABA-inhibited root elongation assay, uniformed two-day-old seedlings were transferred to fresh 1/2 MS plates containing 0, 5 and 10 μM ABA for another seven days vertical growth before taking pictures to measure the length of primary roots following the procedure as described previously [44]. These experiments were repeated three times with similar results.

### 4.4. Subcellular Localization Assays

The possible subcellular localization of ZmFLZs was first predicted using the TargetP-2.0 web server (http://www.cbs.dtu.dk/services/TargetP/; accessed on 15 November 2019) [45]. Nuclear localization of ZmFLZs proteins was predicted by NLStradamus (https://nucpred.bioinfo.se/cgi-bin/single.cgi; accessed on 27 March 2019) [46]. To experimentally analyze the subcellular localization of ZmFLZs and their co-localization with ZmSnRK1α, the cDNAs of the eight *ZmFLZs* were separately cloned into *pCAMBIA1300* vector to in-frame fuse with a *GFP* gene (*ZmFLZ-GFP*), while the cDNA of *ZmSnRK1α* was cloned into *pBI221* vector to fuse with a mCherry tag (*ZmKIN10-mCherry*). Primers used in this study were listed in the Table S2. The plasmid combinations of ZmFLZ-GFP and nuclear marker NLS-mCherry or ZmKIN10-mCherry were introduced into maize leaf protoplasts to determine their subcellular localization. Maize leaf protoplast isolation and PEG-mediated transformation was performed as previously described [47,48]. The transfected protoplast cells were incubated in 1 mL W5 solution (154 mM of NaCl, 125 mM of CaCl_2_, 5 mM of KCl, and 2 mM of MES at pH 5.7) supplemented with 10 mM sucrose for 12 h in the dark at 26 °C before imaging with a laser scanning confocal microscope (LSM800, Zeiss, Jena, Germany).

### 4.5. Yeast-Two-Hybrid Assay

The yeast-two-hybrid assay was carried out following the manufacturer’s instructions (TaKaRa, Beijing, China). The cDNA of the *ZmSnRK1α* was cloned into *pGBKT7* as bait and the cDNAs of the *ZmFLZs* were cloned into *pGADT7* as prey, respectively. Primers used for cloning were listed in Table S2. Different pairs of bait and prey vectors indicated were co-transformed into the yeast reporter strain AH109. Then the different yeast transformants were grown on synthetic double drop-out (SD/-Leu/-Trp) and synthetic triple drop-out (SD/-Leu/-Trp/-Ade) solid medium to examine the protein-protein interaction.

### 4.6. RNA Isolation and Quantitative Real-Time PCR

The samples of maize B73 tissues or organs at specific developmental stages were collected according to the methods as described previously [49]. Total RNA was isolated from the harvested samples using the TransZol Plant kit (TransGen, Beijing, China), then subjected to the cDNA synthesis using the TransScript All-in-One First-Strand cDNA synthesis kit (TaKaRa, Beijing, China) according to the manufacturer’s instructions. Diluted cDNA samples were used for real-time PCR amplification in 10 μL reaction volumes with specific primers and SYBR Green PCR Master Mix (TaKaRa, Beijing, China). Quantitative real-time PCR was run on the Applied Biosystems 7500 system (Prism^®^ 7500, Carlsbad, America) using the following reaction conditions: 95 °C for 1 min followed by 45 cycles of 95 °C for 10 sec and 60 °C for 15 sec. *ZmACTIN* gene was used as the internal control, and the relative expression level of each gene were calculated using the 2^−∆∆CT^ method [50]. Primers used in this study were listed in Table S2.

### 4.7. Statistical Analyses

At least three biological replicates were used during the stress treatments. *t*-test in Graphpad Prism 8 was used to estimate the statistical significance of the difference between the treatment group and the control group and marked with * when *p* < 0.05.

## Data Availability

Data is contained within the article or Supplementary Materials.

## Supplementary Materials

The following are available online at https://www.mdpi.com/article/10.3390/ijms22073529/s1, Supplementary materials including Figures S1–S3 and Tables S1 and S2 can be found online.
