# Supplementary material for: Genome-wide Identification and Characterization of FCS-Like Zinc Finger (FLZ) Family Genes in Maize (Zea mays) and Functional Analysis of ZmFLZ25 in Plant Abscisic Acid Response
Source: Int J Mol Sci. 2021 Mar 29;22(7):3529. doi: 10.3390/ijms22073529 (PMC8037668; doi:10.3390/ijms22073529)
Supplement: Supplementary file 1 [file ijms-22-03529-s001.pdf]

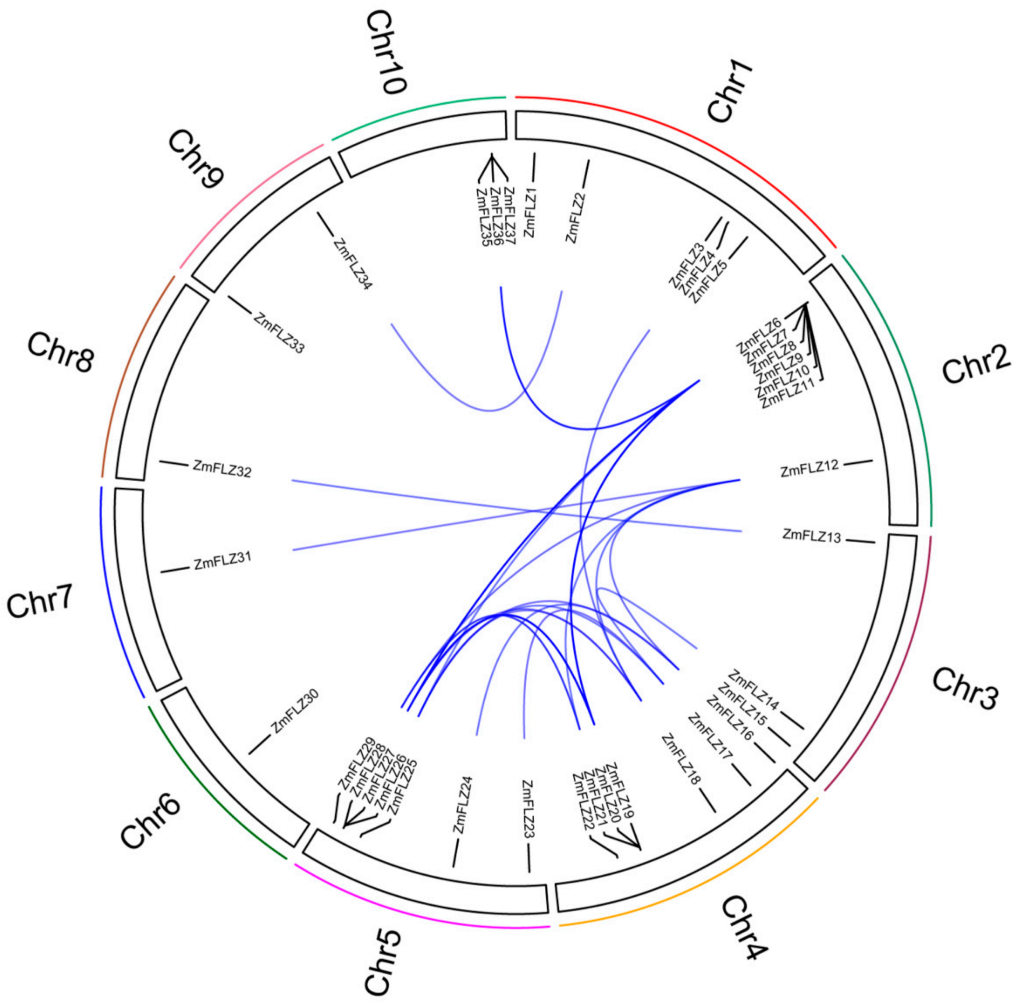

Figure S1. Chromosomal localization and duplication of *ZmFLZs* in maize.

The protein sequences were formatted and aligned to each other with blastp using default parameters (e-value<0.001). The colinearity among the ZmFLZ proteins were identified with MCscanX based on the annotation of B73 reference genome and the aligned results. The results were then visualized with RCircos package in R.

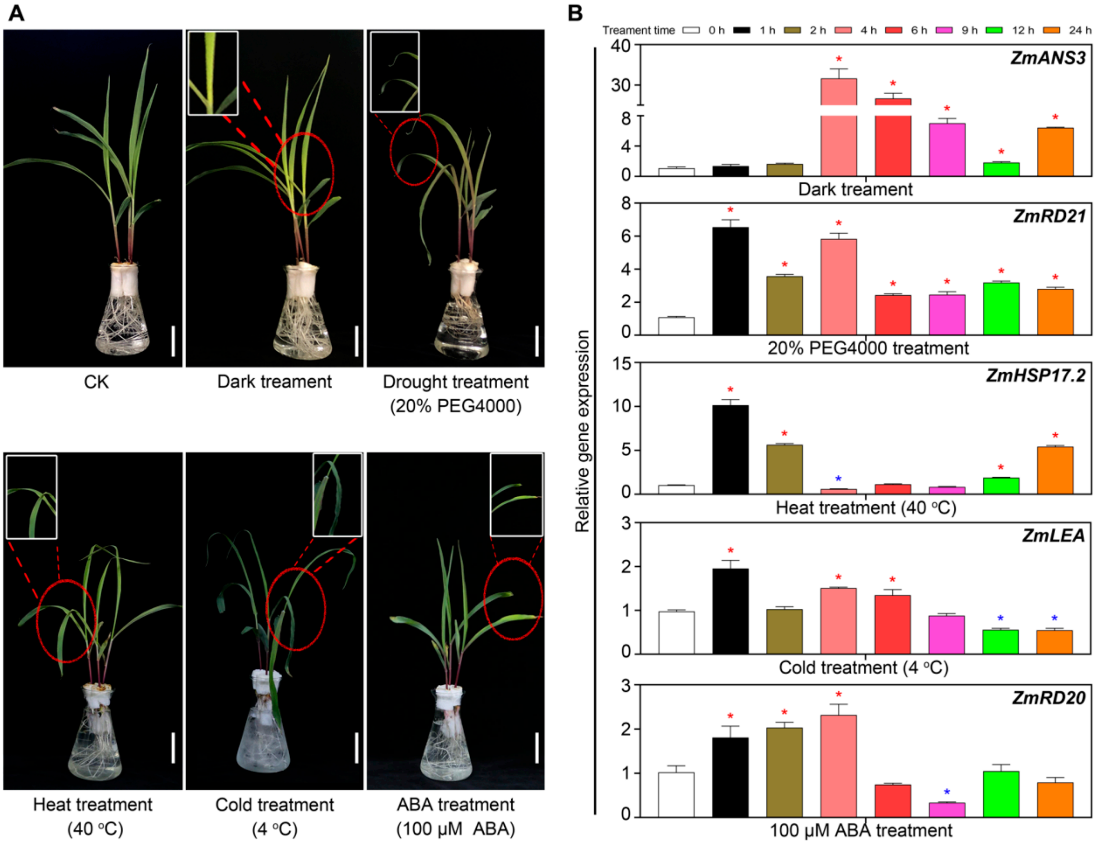

Figure S2. Phenotype of maize seedlings under abiotic stresses and expression patterns of marker genes.

(A) Phenotype of maize Seedlings of B73 were separately subjected to darkness, drought (20% PEG4000), heat (40°C), cold (4°C), and 100 μM ABA treatments. (B) qRT-PCR was used to detect the expression patterns of the marker genes under different stresses. Meterials were harvested at the indicated time points for gene expression analysis by qRT-PCR assay. Seedlings grown under the normal conditions were harvested at the same time courses and used as the controls. Relative gene expression levels of marker genes were calculated as the ratio of “treatment” to “control” after being normalized against *ZmActin*. Data are the mean ± SEM from three independent experiments. The red and blue asterisks indicated significantly enhanced and repressed expression under stresses, respectively (p-value $\leqslant$ 0.05).

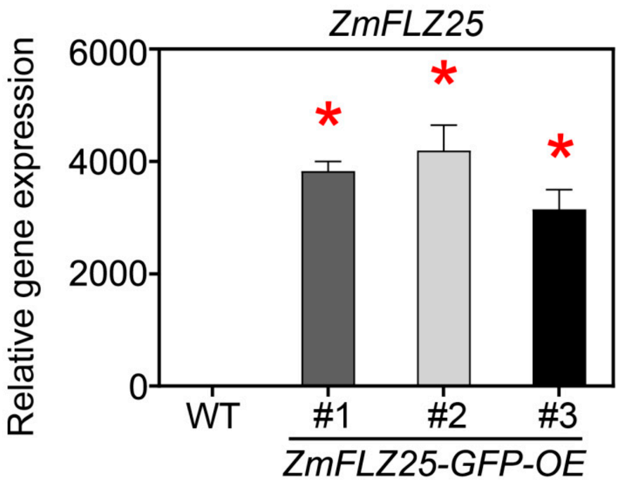

Figure S3. The expression level of *ZmFLZ25-OE* genes in *Arabidopsis*. The *x*-axis represents different plant of *Arabidopsis*. WT: Columbia-0. OE1, OE2, OE3, represents different over expressed of *ZmFLZ25* of *Arabidopsis* lines. The *y*-axis shows the relative gene expression levels normalized to *AtUBQ*. All the data are the mean ± SEM from three independent experiments. The asterisks indicated significantly differences between WT and OE lines as determined by t-test (p-value $\leqslant$ 0.05).

Table S1. Overview of FCS-like zinc finger genes identified in maize.

Gene name[a]	Gene ID<br>Gene locus[b]	Accession number[c]	DNA attributes<br>Ch	ORF (bp)	No. of exons	Protein attributes<br>Length (aa)	MW (kDa)	PI	Localization[d]	NLS predicts[e]
ZmFLZ1	Zm00001d027932	LOC103631809	1	702	2	233	25.71	10.8	Nucl; Chlo	NA
ZmFLZ2	Zm00001d029447	LOC103636115	1	594	1	197	20.71	8.5	Pero; Nucl	NA
ZmFLZ3	Zm00001d032098	LOC100275491	1	573	2	190	21.07	11.3	Cyto; Mito	NA
ZmFLZ4	Zm00001d032292	LOC100272399	1	570	2	189	20.04	5.3	Mito; Cyto	NA
ZmFLZ5	Zm00001d032937	LOC103643562	1	978	2	325	34.92	9.9	Nucl; Chlo	Positive
ZmFLZ6	Zm00001d002520	LOC100274866	2	303	1	100	10.84	7.9	ER	NA
ZmFLZ7	Zm00001d002521	LOC100278820	2	273	1	90	9.8	6.2	Cyto	NA
ZmFLZ8	Zm00001d002522	LOC100275085	2	309	1	102	11.68	5.2	Cyto	NA
ZmFLZ9	Zm00001d002523	LOC100277698	2	387	2	128	15.01	7.5	Cyto	NA
ZmFLZ10	Zm00001d002524	LOC103646017	2	390	2	129	14.93	9.8	Nucleus	Positive
ZmFLZ11	Zm00001d002530	LOC100276205	2	453	2	150	15.28	8.3	Nucl; Mito	NA
ZmFLZ12	Zm00001d005657	LOC103647382	2	534	2	177	18.87	8.6	Mito; Cyto	NA
ZmFLZ13	Zm00001d039597	LOC103643065	3	1077	2	358	36.45	8.1	Cyto	NA
ZmFLZ14	Zm00001d043612	LOC100274019	3	765	2	254	26.67	6.1	Cyto; Mito	NA
ZmFLZ15	Zm00001d044365	LOC100281547	3	795	2	264	29.01	4.4	Chlo; Cyto	NA
ZmFLZ16	Zm00001d048694	LOC100275205	4	489	2	162	17.16	6.5	ER	NA
ZmFLZ17	Zm00001d049577	LOC103655213	4	522	2	173	18.6	6.0	Nucl	NA
ZmFLZ18	Zm00001d050285	LOC100277785	4	558	2	185	19.73	7.3	Mito	NA
ZmFLZ19	Zm00001d051492	LOC103653928	4	486	2	161	18.54	10.2	Mito	NA
ZmFLZ20	Zm00001d051495	LOC103653929	4	468	2	155	17.54	7.8	Nucl; Chlo	Positive
ZmFLZ21	Zm00001d051496	LOC100278490	4	282	2	93	9.76	10.6	Cyto; Mito	NA
ZmFLZ22	Zm00001d052234	LOC100274617	4	465	2	154	16.24	8.7	Nucl; Chlo	NA
ZmFLZ23	Zm00001d013595	LOC100382411	5	330	2	109	11.43	4.8	Cyto	NA
ZmFLZ24	Zm00001d015420	LOC103626680	5	657	2	218	22.93	7.7	Cyto; Chlo	NA
ZmFLZ25	Zm00001d017065	LOC100277951	5	357	2	118	13.36	8.6	Chlo; Cyto	NA
ZmFLZ26	Zm00001d017646	LOC100278632	5	273	2	90	9.6	8.7	Nucl; Mito	NA
ZmFLZ27	Zm00001d017647	LOC103627565	5	459	2	152	17.05	8.1	Nucl	Positive
ZmFLZ28	Zm00001d017648	LOC100279084	5	345	1	114	12.51	8.3	Nucl	NA
ZmFLZ29	Zm00001d018076	LOC103627751	5	486	2	161	16.77	6.5	Nucl; Chlo	NA
ZmFLZ30	Zm00001d036409	LOC100275040	6	396	2	131	14.63	8.3	Nucl	NA
ZmFLZ31	Zm00001d020264	LOC100272233	7	546	2	181	19.11	8.4	Mito	NA
ZmFLZ32	Zm00001d008782	LOC100276685	8	678	2	225	23.53	4.4	Chlo	NA
ZmFLZ33	Zm00001d044849	LOC100276146	9	474	2	157	16.07	9.9	Nucl; Chlo	Positive
ZmFLZ34	Zm00001d047260	LOC100276881	9	576	1	191	19.79	8.9	Pero; Nucl	NA
ZmFLZ35	Zm00001d026026	LOC100273313	10	483	2	160	16.19	8.7	Cyto	Yes
ZmFLZ36	Zm00001d026028	LOC100279111	10	291	1	96	10.82	5.1	Cyto	NA
ZmFLZ37	Zm00001d026029	LOC100502301	10	315	1	104	11.53	8.2	ER	NA

[a]Systematic designation given to maize FCS-like zinc finger genes.

[b]Accession number of locus ID from the MaizeGDB (http://www.maizegdb.org/).

[c]Accession numbers of protein sequence available at NCBI (http:// www.ncbi.nlm.nih.gov/) .

[d]Subcellular localization of ZmFLZs predicted by TargetP-2.0 Server (http://www.cbs.dtu.dk/services/TargetP/).
Ch,chromosome; CDS, coding sequence; No., number; MW, molecular weight; PI, isoelectric point;
Nuc, nuclear; Cyto, cytoplasm; Chl, chloroplast; Mito, mitochondrion.
[e]Nuclear localization of ZmFLZs proteins predicted by NucPred (https://nucpred.bioinfo.se/cgi-bin/single.cgi).
When the nucpred score is greater than 0.6, it will be marked as "Positive", otherwise, it will be marked as "NA".

Table S2. Primers used for gene cloning and yeast-two-hybrid assay:

ZmKIN10-F	CAGTGAATTCCACCCGATGGAGGGAGCGGGAAGAGAT
ZmKIN10-R	TATCGATGCCCACCCTCAAAGAACTCTCAGCTGAG
ZmFLZ5-F	CAGTGAATTCCACCCGATGTTGGGGAGGATGGCTGG
ZmFLZ5-R	TATCGATGCCCACCCTCAGCAGGATTCTCCTTCTT
ZmFLZ9-F	CAGTGAATTCCACCCGATGGCGCACTACCACCCCGT
ZmFLZ9-R	TATCGATGCCCACCCTCACCACGCCCAGATGGGGATCC
ZmFLZ13-F	CAGTGAATTCCACCCGATGCTGCGGAGGATGGTGCC
ZmFLZ13-R	TATCGATGCCCACCCTCACGTCGCGACCACCATGC
ZmFLZ14-F	CAGTGAATTCCACCCGATGGTGGAGTCCAGTGGCCA
ZmFLZ14-R	TATCGATGCCCACCCTCAAAGATTTCGAGCAGGAT
ZmFLZ25-F	CAGTGAATTCCACCCGATGGCCGGGCTAAGCGTTCT
ZmFLZ25-R	TATCGATGCCCACCCTCACGTCTCCCATTCACGTTCCC
ZmFLZ23-F	CAGTGAATTCCACCCGATGCTGCGCAACAGATCGAG
ZmFLZ23-R	TATCGATGCCCACCCCTAACAACGCCCTCCTCCTT
ZmFLZ32-F	CAGTGAATTCCACCCGATGCTGCGGAGGACTGTGCC
ZmFLZ32-R	TATCGATGCCCACCCTCACGTCGCGACCACCATGC
ZmFLZ36-F	CAGTGAATTCCACCCGATGGCCTCCGCCTTCTTCTT
ZmFLZ36-R	TATCGATGCCCACCCTCAGGTCGCCACGGATACCTTCA

Primers used for gene cloning and subcellular localization study:

ZmKIN10-F	GCGCCCGGGATGGAGGGAGCGGGAAGAGATGCC
ZmKIN10-R	GCGGGTACCTCAAAGAACTCTCAGCTGAG
ZmFLZ5-F	CTGATTAACAGGGATCCCCCATGTTGGGGAGGATGGCTGG
ZmFLZ5-R	TCGAGACTAGTGGTACCCCCGCAGGATTCTCCTTCTTCGT
ZmFLZ9-F	CTGATTAACAGGGATCCCCCATGGCGCACTACCACCCCGT
ZmFLZ9-R	TCGAGACTAGTGGTACCCCCCCACGCCCAGATGGGGATCC
ZmFLZ13-F	CTGATTAACAGGGATCCCCCATGCTGCGGAGGATGGTGCC
ZmFLZ13-R	TCGAGACTAGTGGTACCCCCCGTCGCGACCACCATGCCGG
ZmFLZ14-F	CTGATTAACAGGGATCCCCCTGGTGGAGTCCAGTGGCCAC
ZmFLZ14-R	TCGAGACTAGTGGTACCCCCAAGATTTCGAGCAGGATCCA
ZmFLZ23-F	CTGATTAACAGGGATCCCCCATGCTGCGCAACAGATCGAG
ZmFLZ23-R	TCGAGACTAGTGGTACCCCCACAACGCCCTCCTCCTTTTC
ZmFLZ25-F	CTGATTAACAGGGATCCCCCATGGCCGGGCTAAGCGTTCT
ZmFLZ25-R	TCGAGACTAGTGGTACCCCCCGTCTCCCATTCACGTTCCC
ZmFLZ32-F	CTGATTAACAGGGATCCCCCATGCTGCGGAGGACTGTGCC
ZmFLZ32-R	TCGAGACTAGTGGTACCCCCCGTCGCGACCACCATGCCGG
ZmFLZ36-F	CTGATTAACAGGGATCCCCC ATGGCCTCCGCCTTCTTCTT
ZmFLZ36-R	TCGAGACTAGTGGTACCCCC GGTCGCCACGGATACCTTCA

Primers used for qRT-PCR:

ZmActin-F	ATGGTCAAGGCCGGTTTCG
ZmActin-R	TCAGGATGCCTCTCTTGGCC
semi-Zmactin-F	AACCATCCCTTGTTGGCATG
semi-Zmactin-R	AGCAAACTCTGAGGCAACAC

ZmFLZ5-qPCR-F	TGTCGCCGAGGAAGATGATG
ZmFLZ5-qPCR-R	TCGAATATGTGCGTCGTCCTC
ZmFLZ9-qPCR-F	ACTCCTCCTCCCCTCTGAAG
ZmFLZ9-qPCR-R	AAGCAGATGTCGAGGTAGTGG
ZmFLZ10-qPCR-F	TCGGCCTCTCGTATTTTGGC
ZmFLZ10-qPCR-R	AGTCGATCTGCTGGTAGGAT
ZmFLZ13-qPCR-F	CGTGCAAGAAGAAGCTTGAAGG
ZmFLZ13-qPCR-R	TTGTTCTCGGCTTCCTCCTC
ZmFLZ14-qPCR-F	TCGACTCCATCAAGCCGTTC
ZmFLZ14-qPCR-R	AAGTGTACTCCTCGCAGTCG
ZmFLZ16-qPCR-F	ACATGTACAGAGGCGAGAGG
ZmFLZ16-qPCR-R	CCCGACCGAGGTAGTAGTGT
ZmFLZ18-qPCR-F	GGAGTGGAAGGAGAAGTGCG
ZmFLZ18-qPCR-R	TAGGACTTGGAGGCTAGGACC
ZmFLZ20-qPCR-F	AACAGCTTCCTGGACGAGTG
ZmFLZ20-qPCR-R	CTTCTTCCTGCGGTGCCTTA
ZmFLZ25-qPCR-F	CCTGTGCCACAAAGAGCTTG
ZmFLZ25-qPCR-R	TCCTCGTCCATGAAGATCTGC
ZmFLZ32-qPCR-F	AAGAGGAAGCCGAGAACAGC
ZmFLZ32-qPCR-R	AAGATGTCGTCGTCGTGGAAG
ZmFLZ36-qPCR-F	CCTCCGCCTTCTTCTTCGAC
ZmFLZ36-qPCR-R	ACATGAAGATGTCGCAGTCG
ZmKIN10-qPCR-F	TGGCAAAACCCTGGGAATTG
ZmKIN10-qPCR-R	CCATGCTTCTGATCTTCTTGCG
ZmASN3-qPCR-F	ACTGGTCTCAGGCAAAGAGG
ZmASN3-qPCR-R	TTGCCCTCGTGCTGGTAGAG
ZmRD20-qPCR-F	CTCCTTTGCTGTCCATCCGT
ZmRD20-qPCR-R	TGAGCTCAACTCGTCTTTTGTT
ZmRD21-qPCR-F	TGTCGATCGTGTCCTACGGG
ZmRD21-qPCR-R	CGTCGATGTAGCGGAGGTTG
ZmLEA-qPCR-F	GTCGAGGACGCGATGTACC
ZmLEA-qPCR-R	ATTCTCCCCTTGACCGGGAT
ZmHSP17.2-qPCR-F	AAGAAGCCTGAGGTGAAGGC
ZmHSP17.2-qPCR-R	GACCAGCAGCAGTACACCTT
